# [Retracted] Hsp70 inducer, 17-allylamino-demethoxygeldanamycin, provides neuroprotection via anti-inflammatory effects in a rat model of traumatic brain injury

**DOI:** 10.3892/etm.2026.13291

**Published:** 2026-09-10

**Authors:** Youquan Gu, Jun Chen, Tianhong Wang, Chaoning Zhou, Zhaodong Liu, Lanhua Ma

Exp Ther Med 12:3767–3772, 2016; DOI: 10.3892/etm.2016.3821

Following the publication of the above paper, it was drawn to the Editor's attention by a concerned reader that, in addition to the apparent duplication of data within Figs. 4A, 4B and 6B, and Figs. 6A and 8A in the paper itself, western blot data featured in Figs. 6A, 6B and 8A were strikingly similar to data appearing in different form in other articles written by different authors at different research institutes that had either already been submitted for publication, or were already published, before the submission of this paper to *Experimental and Therapeutic Medicine*.

In view of the fact that the abovementioned data had already apparently been published before this article was received at *Experimental and Therapeutic Medicine*, the Editor has decided that this paper should be retracted from the Journal. The authors were asked for an explanation to account for these concerns, but the Editorial Office did not receive a reply. The Editor apologizes to the readership for any inconvenience caused.

